# 4,4′-Bis(trimethyl­sil­yl)-2,2′-bipyridine

**DOI:** 10.1107/S1600536811043704

**Published:** 2011-11-02

**Authors:** Chang-Ge Zheng, Hua-Peng Cao, Yang Song

**Affiliations:** a School of Chemical and Material Engineering, Jiangnan University, 1800 Lihu Road, Wuxi, Jiangsu Province 214122, People’s Republic of China

## Abstract

In the mol­ecule of title compound, C_16_H_24_N_2_Si_2_, the pyridine rings are nearly planar (r.m.s. deviation = 0.002 Å).

## Related literature

For the structure of 5,5′-bis­(trimethyl­sil­yl)-2,2′-bipyridines, see: Stange *et al.* (2000 ▶). For the structure of 4-trimethyl­silyl­pyridine, see: Postigo & Rossi (2001 ▶). For synthetic procedure to obtain 4,4′-bis­(methox­yl)-2,2′-bipyridine, see: Wenkert & Woodward (1983 ▶).
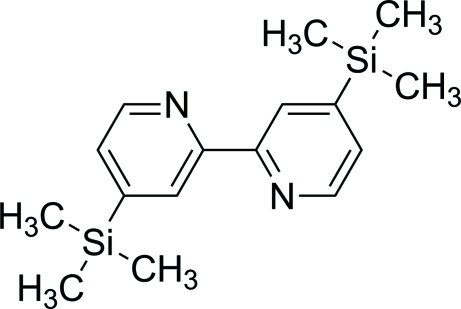


## Experimental

### 

#### Crystal data

C_16_H_24_N_2_Si_2_
*M* *_r_* = 300.55
Monoclinic, 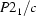

*a* = 13.154 (4) Å
*b* = 6.4599 (16) Å
*c* = 11.280 (3) Å
β = 111.222 (6)°
*V* = 893.5 (4) Å^3^
*Z* = 2
Mo *K*α radiation
μ = 0.19 mm^−1^
*T* = 223 K
0.50 × 0.30 × 0.20 mm

#### Data collection

Rigaku Saturn CCD diffractometer
Absorption correction: multi-scan (*SADABS*; Sheldrick, 1996 ▶) *T*_min_ = 0.869, *T*_max_ = 0.963
4311 measured reflections
1649 independent reflections
1364 reflections with *I* > 2σ(*I*)
*R*_int_ = 0.027

#### Refinement

*R*[*F* ^2^ > 2σ(*F* ^2^)] = 0.046
*wR*(*F* ^2^) = 0.125
*S* = 1.08
1649 reflections
95 parameters
2 restraints
H-atom parameters constrained
Δρ_max_ = 0.25 e Å^−3^
Δρ_min_ = −0.26 e Å^−3^

### 

Data collection: *CrystalClear* (Rigaku, 2005 ▶); cell refinement: *CrystalClear*; data reduction: *CrystalClear*; program(s) used to solve structure: *SHELXS97* (Sheldrick, 2008 ▶); program(s) used to refine structure: *SHELXL97* (Sheldrick, 2008 ▶); molecular graphics: *PLATON* (Spek, 2009 ▶); software used to prepare material for publication: *SHELXL97*.

## Supplementary Material

Crystal structure: contains datablock(s) global, I. DOI: 10.1107/S1600536811043704/rk2303sup1.cif

Structure factors: contains datablock(s) I. DOI: 10.1107/S1600536811043704/rk2303Isup2.hkl

Supplementary material file. DOI: 10.1107/S1600536811043704/rk2303Isup3.cml

Additional supplementary materials:  crystallographic information; 3D view; checkCIF report

## Figures and Tables

**Fig. 1.**
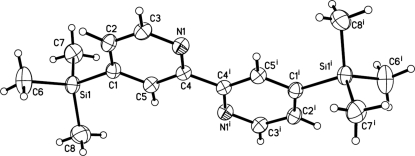
Molecular structure of title compound with the atom numbering scheme. Displacement ellipsoids are drawn at 40% probability level. H atoms are presented as a small spheres of arbitrary radius. Symmetry code: (i) -x + 1, -y + 1, -z.

## supplementary crystallographic information

### Comment

Derivatives of 2,2'-bipyridine have received much attention due to their potential to form polypyridyl metal complexes, particularly of ruthenium and rhenium which have diverse applications. The photochemical and redox properties of these complexes can be varied through appropriate substitution on the pyridine rings. The derivatization of a 2,2'-bipyridine ligand with electron donating/withdrawing groups in the 4,4'-positions has been a popular means of controlling the redox potential of transition metal bipyridine complexes. The 4,4'-disubstitution can also offers no steric complications on complexation. The research about synthesis and properties of pyridine rings interconnected with strong electron-donating groups, as well as trimethysilyl group, was recently reported (Stange *et al.*, 2000; Postigo & Rossi, 2001). However, there are no reports on 4,4'-bis(trimethylsilyl)-2,2'-bipyridine. Herein, we report crystal structure of the title compound.

The molecule is placed in centre of symmetry and nealy flat (Fig. 1) as the C—Si is co-planar with the aromatic rings. The torsion angle for N1—C4—C4^i^—C5^i^ = 0°. In crystal, molecules are connected by weak non-classical intermolecular C3–H3···N1^ii^ hydrogen bonds with parameters C3···N1^ii^ = 3.626 (2) Å, H3···N1^ii^ = 2.714 Å and angle C3–H3···N1^ii^ = 164.3°. Symmetry codes: (i) -*x* + 1, -*y* + 1, -*z*; (ii) -*x* + 1, *y* - 1/2, -*z* + 1/2.

### Experimental

All the reagents and solvents empolyed were commercially available. The title compound was synthesized by using 2,2'-bipyridine as the starting material with successive polystepreactions (Wenkert & Woodward, 1983). The final product was dissolved in the solution of methanol and methylene chloride, which diffused slowly. After seven days, colourless block-shaped crystals were obtained which were suitable for X-ray analysis.

### Refinement

All H atoms were placed in geometrically idealized positions. H atoms of bipyridine constrained to ride on their parent atoms with C—H = 0.94 Å and refined with *U*_iso_(H) = 1.2*U*_iso_(C). The H atoms of methyl groups constrained to ride on their parent atoms with C—H = 0.97 Å and refined with *U*_iso_(H) = 1.5*U*_iso_(C).

### Crystal data

**Table:**

C_16_H_24_N_2_Si_2_	*F*(000) = 324
*M**_r_* = 300.55	*D*_x_ = 1.117 Mg m^−^^3^
Monoclinic, *P*2_1_/*c*	Mo *K*α radiation, λ = 0.71075 Å
Hall symbol: -P 2ybc	Cell parameters from 3568 reflections
*a* = 13.154 (4) Å	θ = 3.2–27.5°
*b* = 6.4599 (16) Å	µ = 0.19 mm^−^^1^
*c* = 11.280 (3) Å	*T* = 223 K
β = 111.222 (6)°	Block, colourless
*V* = 893.5 (4) Å^3^	0.50 × 0.30 × 0.20 mm
*Z* = 2	

### Data collection

**Table:**

Rigaku Saturn CCD diffractometer	1649 independent reflections
Radiation source: fine-focus sealed tube	1364 reflections with *I* > 2σ(*I*)
graphite	*R*_int_ = 0.027
Detector resolution: 14.63 pixels mm^-1^	θ_max_ = 25.5°
ω scans	*h* = −13→15
Absorption correction: multi-scan (*SADABS*; Sheldrick, 1996)	*k* = −7→7
*T*_min_ = 0.869, *T*_max_ = 0.963	*l* = −13→12
4311 measured reflections	

### Refinement

**Table:**

Refinement on *F*^2^	Primary atom site location: structure-invariant direct methods
Least-squares matrix: full	Secondary atom site location: difference Fourier map
*R*[*F*^2^ > 2σ(*F*^2^)] = 0.046	Hydrogen site location: inferred from neighbouring sites
*wR*(*F*^2^) = 0.125	H-atom parameters constrained
*S* = 1.08	*w* = 1/[σ^2^(*F*_o_^2^) + (0.0734*P*)^2^ + 0.0832*P*] where *P* = (*F*_o_^2^ + 2*F*_c_^2^)/3
1649 reflections	(Δ/σ)_max_ < 0.001
95 parameters	Δρ_max_ = 0.25 e Å^−^^3^
2 restraints	Δρ_min_ = −0.26 e Å^−^^3^

### Special details

**Table:**

Geometry. All s.u.'s (except the s.u. in the dihedral angle between two l.s. planes) are estimated using the full covariance matrix. The cell s.u.'s are taken into account individually in the estimation of s.u.'s in distances, angles and torsion angles; correlations between s.u.'s in cell parameters are only used when they are defined by crystal symmetry. An approximate (isotropic) treatment of cell s.u.'s is used for estimating s.u.'s involving l.s. planes.
Refinement. Refinement of *F*^2^ against ALL reflections. The weighted *R*-factor *wR* and goodness of fit *S* are based on *F*^2^, conventional *R*-factors *R* are based on *F*, with *F* set to zero for negative *F*^2^. The threshold expression of *F*^2^ > σ*F*^2^ is used only for calculating *R*-factors(gt) *etc*. and is not relevant to the choice of reflections for refinement. *R*-factors based on *F*^2^ are statistically about twice as large as those based on *F*, and *R*-factors based on ALL data will be even larger.

### Fractional atomic coordinates and isotropic or equivalent isotropic displacement parameters (Å^2^)

**Table:**

	*x*	*y*	*z*	*U*_iso_*/*U*_eq_	
Si1	0.80366 (4)	0.08423 (9)	0.02023 (5)	0.0405 (2)	
N1	0.49321 (13)	0.2932 (2)	0.10461 (14)	0.0366 (4)	
C1	0.68120 (15)	0.1675 (3)	0.05735 (17)	0.0356 (5)	
C2	0.64022 (16)	0.0538 (3)	0.13516 (18)	0.0383 (5)	
H2	0.6754	−0.0686	0.1740	0.046*	
C3	0.54791 (17)	0.1206 (3)	0.15535 (18)	0.0391 (5)	
H3	0.5221	0.0400	0.2079	0.047*	
C4	0.53132 (15)	0.4054 (3)	0.02861 (17)	0.0311 (4)	
C5	0.62411 (16)	0.3477 (3)	0.00480 (17)	0.0353 (5)	
H5	0.6487	0.4314	−0.0475	0.042*	
C6	0.8938 (2)	−0.0751 (5)	0.1542 (3)	0.0724 (9)	
H6A	0.8573	−0.2038	0.1584	0.109*	
H6B	0.9101	0.0003	0.2333	0.109*	
H6C	0.9611	−0.1049	0.1408	0.109*	
C7	0.7569 (2)	−0.0682 (4)	−0.1293 (2)	0.0576 (6)	
H7A	0.8197	−0.1205	−0.1457	0.086*	
H7B	0.7140	0.0195	−0.1993	0.086*	
H7C	0.7127	−0.1834	−0.1210	0.086*	
C8	0.8778 (2)	0.3196 (4)	0.0009 (3)	0.0687 (7)	
H8A	0.8948	0.4060	0.0760	0.103*	
H8B	0.8323	0.3964	−0.0732	0.103*	
H8C	0.9448	0.2790	−0.0101	0.103*	

### Atomic displacement parameters (Å^2^)

**Table:**

	*U*^11^	*U*^22^	*U*^33^	*U*^12^	*U*^13^	*U*^23^
Si1	0.0418 (4)	0.0433 (4)	0.0397 (4)	0.0085 (2)	0.0189 (3)	0.0001 (2)
N1	0.0437 (9)	0.0376 (9)	0.0332 (8)	0.0016 (7)	0.0195 (7)	0.0033 (7)
C1	0.0387 (10)	0.0352 (10)	0.0323 (9)	0.0035 (8)	0.0120 (8)	−0.0037 (8)
C2	0.0451 (11)	0.0361 (10)	0.0339 (10)	0.0059 (9)	0.0144 (9)	0.0028 (8)
C3	0.0496 (12)	0.0373 (11)	0.0347 (10)	−0.0001 (9)	0.0202 (9)	0.0051 (8)
C4	0.0368 (10)	0.0307 (9)	0.0277 (9)	−0.0011 (8)	0.0139 (8)	−0.0013 (7)
C5	0.0410 (10)	0.0377 (10)	0.0317 (9)	0.0005 (8)	0.0185 (8)	−0.0008 (8)
C6	0.0688 (17)	0.093 (2)	0.0590 (15)	0.0392 (15)	0.0270 (14)	0.0160 (14)
C7	0.0642 (15)	0.0625 (15)	0.0532 (13)	0.0052 (12)	0.0297 (12)	−0.0119 (11)
C8	0.0544 (14)	0.0656 (16)	0.097 (2)	−0.0045 (13)	0.0409 (14)	−0.0089 (15)

### Geometric parameters (Å, °)

**Table:**

Si1—C7	1.855 (2)	C4—C4^i^	1.485 (3)
Si1—C6	1.858 (2)	C5—H5	0.9400
Si1—C8	1.861 (3)	C6—H6A	0.9700
Si1—C1	1.884 (2)	C6—H6B	0.9700
N1—C3	1.338 (2)	C6—H6C	0.9700
N1—C4	1.350 (2)	C7—H7A	0.9700
C1—C2	1.394 (3)	C7—H7B	0.9700
C1—C5	1.396 (3)	C7—H7C	0.9700
C2—C3	1.383 (3)	C8—H8A	0.9700
C2—H2	0.9400	C8—H8B	0.9700
C3—H3	0.9400	C8—H8C	0.9700
C4—C5	1.392 (3)		
			
C7—Si1—C6	110.44 (13)	C4—C5—H5	119.5
C7—Si1—C8	110.03 (13)	C1—C5—H5	119.5
C6—Si1—C8	109.91 (14)	Si1—C6—H6A	109.5
C7—Si1—C1	108.97 (10)	Si1—C6—H6B	109.5
C6—Si1—C1	108.84 (11)	H6A—C6—H6B	109.5
C8—Si1—C1	108.60 (10)	Si1—C6—H6C	109.5
C3—N1—C4	116.95 (17)	H6A—C6—H6C	109.5
C2—C1—C5	115.83 (18)	H6B—C6—H6C	109.5
C2—C1—Si1	123.10 (15)	Si1—C7—H7A	109.5
C5—C1—Si1	121.05 (15)	Si1—C7—H7B	109.5
C3—C2—C1	120.11 (18)	H7A—C7—H7B	109.5
C3—C2—H2	119.9	Si1—C7—H7C	109.5
C1—C2—H2	119.9	H7A—C7—H7C	109.5
N1—C3—C2	123.92 (18)	H7B—C7—H7C	109.5
N1—C3—H3	118.0	Si1—C8—H8A	109.5
C2—C3—H3	118.0	Si1—C8—H8B	109.5
N1—C4—C5	122.12 (17)	H8A—C8—H8B	109.5
N1—C4—C4^i^	116.3 (2)	Si1—C8—H8C	109.5
C5—C4—C4^i^	121.6 (2)	H8A—C8—H8C	109.5
C4—C5—C1	121.06 (18)	H8B—C8—H8C	109.5
			
C7—Si1—C1—C2	92.79 (18)	C4—N1—C3—C2	0.6 (3)
C6—Si1—C1—C2	−27.7 (2)	C1—C2—C3—N1	−0.4 (3)
C8—Si1—C1—C2	−147.35 (18)	C3—N1—C4—C5	−0.9 (3)
C7—Si1—C1—C5	−85.66 (18)	C3—N1—C4—C4^i^	179.05 (18)
C6—Si1—C1—C5	153.85 (17)	N1—C4—C5—C1	1.1 (3)
C8—Si1—C1—C5	34.20 (19)	C4^i^—C4—C5—C1	−178.86 (19)
C5—C1—C2—C3	0.5 (3)	C2—C1—C5—C4	−0.9 (3)
Si1—C1—C2—C3	−178.01 (14)	Si1—C1—C5—C4	177.70 (14)

Symmetry codes: (i) −*x*+1, −*y*+1, −*z*.

### Hydrogen-bond geometry (Å, °)

**Table:**

*D*—H···*A*	*D*—H	H···*A*	*D*···*A*	*D*—H···*A*
C3—H3···N1^ii^	0.94	2.71	3.626 (2)	164.

Symmetry codes: (ii) −*x*+1, *y*−1/2, −*z*+1/2.

## References

[bb1] Postigo, A. & Rossi, R. A. (2001). *Org. Lett.* **3**, 1197-1200. 10.1021/ol015666s 11348193

[bb2] Rigaku (2005). *CrystalClear.* Rigaku Corporation, Tokyo, Japan.

[bb3] Sheldrick, G. M. (1996). *SADABS* University of Göttingen, Germany.

[bb4] Sheldrick, G. M. (2008). *Acta Cryst.* A**64**, 112–122. 10.1107/S0108767307043930 18156677

[bb5] Spek, A. L. (2009). *Acta Cryst.* D**65**, 148–155. 10.1107/S090744490804362X PMC2631630 19171970

[bb6] Stange, A. F., Tokura, S. & Kira, M. (2000). *J. Organomet. Chem.* **612**, 117-124.

[bb7] Wenkert, D. & Woodward, R. B. (1983). *J. Org. Chem.* **48**, 283-289.

